# Exploring the Challenges of Implementing a Web-Based Telemonitoring Strategy for Teenagers With Inflammatory Bowel Disease: Empirical Case Study

**DOI:** 10.2196/11761

**Published:** 2019-03-29

**Authors:** Alie Dijkstra, Anke Heida, Patrick Ferry van Rheenen

**Affiliations:** 1 Department of Pediatric Gastroenterology, Hepatology and Nutrition University of Groningen University Medical Centre Groningen Groningen Netherlands; 2 Department of General Practice University of Groningen University Medical Centre Groningen Groningen Netherlands

**Keywords:** eHealth, inflammatory bowel disease, health care improvement, implementation science, quality of care

## Abstract

**Background:**

We designed a telemonitoring strategy for teenagers with inflammatory bowel disease to prevent an anticipated disease flare and avert unplanned office visits and day care procedures. The strategy was evaluated in a randomized controlled trial that involved 11 Dutch pediatric gastroenterology centers, each using repeated symptom scores and stool calprotectin measurements. In the telemonitoring arm of the trial, teenagers (n=84) as well as their health providers were alerted to out-of-range results, and suggestions for change in therapy were offered. We demonstrated that the technology was a safe and cost saving alternative to health checks by the specialist at fixed intervals.

**Objective:**

The aim of this study was to evaluate whether we could move our telemonitoring strategy from a demonstration project to one that is sustained within existing sites.

**Methods:**

In this empirical case study, we used the nonadoption, abandonment, scale-up, spread, and sustainability (NASSS) framework to explore the challenges to implementing our strategy. The framework distinguishes 7 domains: (1) the illness, (2) the technology, (3) the value proposition, (4) the adopter system, (5) the organization, (6) the societal system, and (7) the time dimension. We summarized the challenges across all 7 domains and classified them as simple (+++), complicated (++), or complex (+). Technologies in which multiple domains are complicated have proven difficult to implement, whereas those with multiple complex domains may not even become mainstreamed.

**Results:**

The technology that we used and the linked program (*IBD-live*) allowed us to select and target the teenagers who were most likely to benefit from a face-to-face encounter with their specialist (+++). The value proposition of the technology was clear, with a distinct benefit for patients and an affordable service model, but health providers had plausible personal reasons to resist (double data entry, ++). The organization was not yet ready for the innovation, as it requires a shift to new ways of working (+). We had no concerns about reimbursement, as Dutch health insurers agreed that screen-to-screen consultations will be reimbursed at a rate equivalent to face-to-face consultations (+++). Finally, the technology was considered easy to adapt and evolve over time to meet the needs of its users (+++).

**Conclusions:**

The challenges to be addressed are merely *complicated* (++) rather than *complex* (+), which means that our program may be difficult but not impossible to sustain within existing sites. After integrating the technology and its use with local workflows first, we believe that our telemonitoring strategy will be ready for sustained adoption. In contrast with what we did ourselves, we recommend others to use the NASSS framework prospectively and in real time to predict and explore the challenges to implementing new technologies.

## Introduction

Crohn's disease and ulcerative colitis are the 2 main forms of inflammatory bowel disease (IBD). Both are chronic lifelong conditions that cause intestinal inflammation. The inflammation waxes and wanes over time in a seemingly unpredictable fashion. The prevalence of IBD exceeds 0.3% in North America, Oceania, and many countries in Europe [1]. A third of patients experience at least one flare per year, which is associated with pain, disruption of normal activities, and reduction of quality of life [2].

Follow-up of patients with IBD traditionally comprises scheduled visits regardless of how well the patients feel. For every scheduled visit that is instrumental in preventing unnecessary pain or disability, many more will be uninformative. Selecting and targeting the patients who are most likely to benefit from a face-to-face encounter with their specialist are logical solutions.

We have recently shown that repeated quantitative measurements of fecal calprotectin help specialists and patients recognize disease flares at an early stage [3]. Monitoring calprotectin values longitudinally enables timely adjustment of therapy plans. In modern-day IBD care, the treatment target is to keep calprotectin values below 250 μg/g, which is the equivalent of disease remission [4]. When calprotectin values exceed this critical threshold, the patient has a high risk of disease flare and treatment intensification is justified. Conversely, when calprotectin values drop again below the critical threshold, induction therapy can be safely tapered down.

Although it seems that without monitoring, patients with IBD would not be in a position to receive optimal care, there are several potential difficulties and unresolved matters that relate to the use of calprotectin. It may disrupt doctor-patient communication, unduly focusing attention on inflammatory markers (medicalization and creating worried well), increase health care costs, and bears the risk of overdiagnosing flares as calprotectin may also rise in bacterial gastrointestinal infections.

Between June 2013 and January 2016, we conducted a randomized controlled trial (RCT) comparing a Web-based telemonitoring strategy for teenagers with IBD (*IBD-live*, designed for early detection of relapse) and conventional follow-up [5]. We concluded that patients in the telemonitoring group followed a similar disease course as those in conventional follow-up, although they had significantly less face-to-face encounters with their specialist. Moreover, the number of times blood was taken, trips to the hospital, and hours of absence from school or social activities were lower in the telemonitoring group. The majority of patients assigned to the telemonitoring arm took an active interest in their readings, engaged enthusiastically with the feedback, and found this technical innovation reassuring. In a patient satisfaction survey that was performed after the close-out visit (after 52 weeks of participation), the majority of respondents agreed that telemonitoring was time saving, increased their understanding of the disease, and did not disturb them. Overall, 81% of respondents wished to use the telemonitoring services in future.

We sought to evaluate whether we could move our telemonitoring strategy from a demonstration project to one that is sustained within existing sites.

## Methods

### Context

The Web-based telemonitoring strategy was tested in 11 pediatric gastroenterology practices, which were recruited on the basis of their interest in IBD. The principal investigators at the various sites were pediatric gastroenterologist and members of the *Kids with Crohn’s and Colitis* working group for Collaborative Research in the Netherlands. Together they treat about two-thirds of the total pediatric IBD population in the Netherlands. National treatment guidelines provided uniformity in treatment among practices [6]. Table 1 summarizes practice characteristics for the 11 sites.

### Outline of the Telemonitoring Strategy

**Figure 1:**
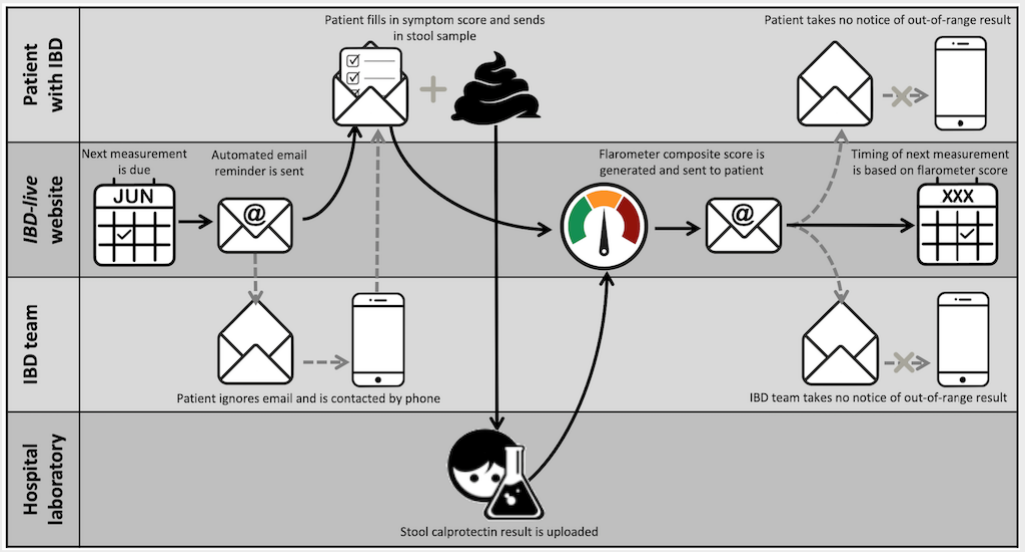
Swim lane process diagram showing the sequence of events from sending an automated reminder by email to the advice on treatment and timing of re-measurement. The parallel lines divide the diagram into lanes, with one lane for each person or subprocess. The horizontal direction represents the sequence of events in the overall process. Arrows between the lanes represent how information or material is passed among the subprocesses. Black arrows represent the ideal flow; gray dotted arrows represent the weakest links in the chain of events. IBD: inflammatory bowel disease.

**Figure 2:**
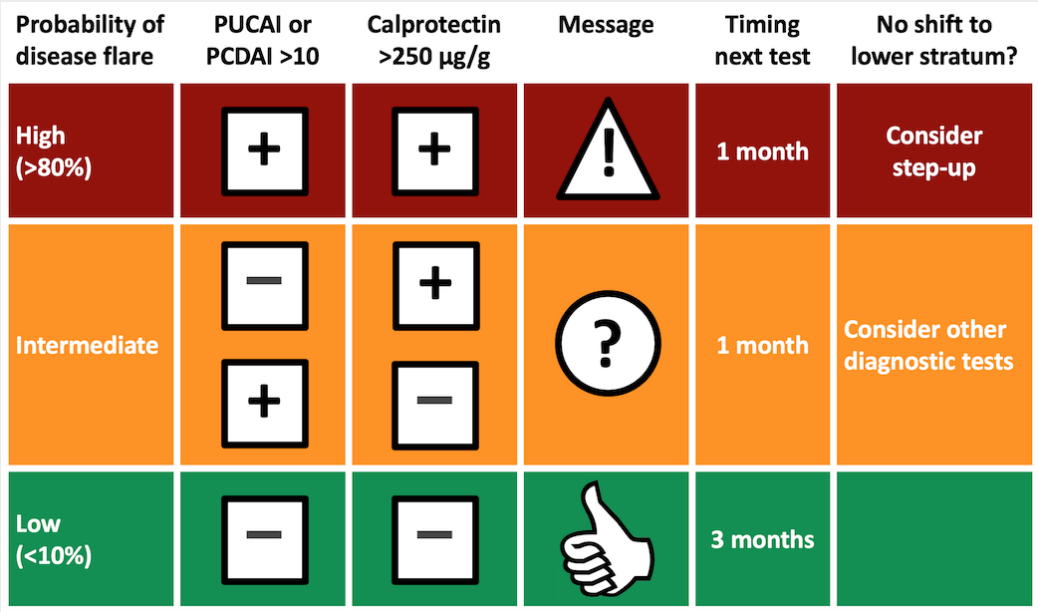
Flarometer strategy. Algorithm with advice on treatment and the timing of re-measurement (figure printed with permission from Heida et al [5]). PUCAI: pediatric ulcerative colitis activity index; PCDAI: pediatric Crohn's disease activity index.

Patients in the telemonitoring group had access to the IBD-live website, a cloud-based portal with 3 modules, as follows: (1) the Flarometer, an automatic cumulation of patient-rated symptom scores and fecal calprotectin measurements, (2) a platform for direct communication with the local IBD team with guaranteed feedback within 2 working days, and (3) a module with study questionnaires (on quality of life, absenteeism, and health care utilization). Patients could freely explore the website, but the majority only accessed it after receiving an email notification that it was time for the next Flarometer measurement (Figure 1). In case the Flarometer was not used despite 3 email notifications with 7-day intervals, the IBD-nurse or specialist phoned the patients to encourage them to fill in the Flarometer and send in a stool sample. The email notification had a link that took the user in a few steps in sequential order to the Flarometer (tunnel design) [7]. The Flarometer strategy is presented in Figure 2. Patients as well as their specialists were alerted to out-of-range results, and suggestions for change in therapy were offered.

**Table 1:** Practice characteristics (N=11).

Characteristics		Number of practices, n
**Practice type**		
	Tertiary care hospital	6
	Large regional general hospital	5
**Practice size**		
	10 to 100 pediatric IBD^a^ patients	7
	More than 100 pediatric IBD patients	4
**Adolescents transitioned to adult-oriented care**		
	More than 10 per year	7
	≥10 per year	4
**Pediatric IBD nurse present**		
	Yes	8
	No	3
**Pediatric endoscopy unit available**		
	Yes	11
	No	0

^a^IBD: Inflammatory bowel disease.

### Evaluation

During the RCT, we gathered information on its potential for implementation in a real-world setting. Data sources included study parameters as specified in the study protocol [8] and ethics paperwork, the log of technical failures, and 1 additional semistructured interview for all patient participants. In addition, for the purpose of this case study, we collected information from 5 research staff including the principal investigator (PFvR) and 2 research coordinators (AD and AH), 13 clinical staff involved in the trial, 4 clinical staff not involved in the trial, 2 hospital decision makers, and 1 Web designer. This information was extracted from 15 semistructured and 4 narrative interviews.

We used the recently published nonadoption, abandonment, scale-up, spread, and sustainability (NASSS) framework to systematically explore the challenges and decide whether we should abandon our telehealth technology or aim to achieve sustained adoption within the existing sites [9].

The NASSS framework comprises measures categorized in 7 domains: (1) the illness, (2) the technology, (3) the value proposition, (4) the adopter system (professional staff, patients, and lay carers), (5) the organization, (6) the wider (institutional and societal) system, and (7) interaction and mutual adaptation among all these domains over time. In Table 2, we summarize all the original measures and how we translated them to assess local applicability. All measures were classified as simple (straightforward, predictable, and few components—as in making a sandwich), complicated (remains predictable, but with multiple interacting components or issues—as in building a rocket), or complex (dynamic, unpredictable, and not easily disaggregated into constituent components—as in raising a child). The choice for 1 of 3 classes was further substantiated by tabulated examples in the NASSS index paper [9].

Technologies in which multiple domains are complicated have proven difficult, slow, and expensive to implement. Those in which multiple domains are complex may not even become mainstreamed.

Overall, 2 assessors (AD, PFvR) independently rated the measures with the use of a predefined matrix. Disagreements were resolved by discussion and, if necessary, a third independent assessor (AH).

### Ethical Considerations

The patient-related data in this paper as well as the log of technical failures were collected as part of the initial RCT. The Medical Ethical Committee of the University Medical Centre Groningen approved the study protocol (METC 2013/010). Secondary approval was obtained from all participating centers. Use of additional data collected from nonpatient participants (including semistructured interviews with specialists and IBD nurses and qualitative interviews with managers and Web designer) in this empirical case study did not require approval according to the Dutch Medical Research Involving Human Subject Act.

**Table 2:** Overview of the nonadoption, abandonment, scale-up, spread, and sustainability (NASSS) framework used to evaluate the success of the *IBD-live* telemonitoring strategy. The left column shows the original NASSS questions, the right column shows how we adapted some to assess local applicability.

Domain and questions		Adapted questions
**Domain 1: The illness**		
	1A. What is the nature of the condition or illness?	How well does the Flarometer strategy predict out-of-range or all-is-well events?
	1B. What are the relevant sociocultural factors and comorbidities?	What are the sociocultural factors associated with good adherence to the technology? (Original RCT^a^)^b^
**Domain 2: The technology**		
	2A. What are the key features of the technology?	No changes
	2B. What kind of knowledge does the technology bring into play?	No changes
	2C. What knowledge and/or support is required to use the technology?	No changes
	2D. What is the technology supply model?	No changes
**Domain 3: The value proposition**		
	3A. What is the developer’s business case for the technology (supply-side value)?	No changes
	3B. What is the desirability, efficacy, safety, and cost effectiveness of the technology (demand-side value)?	What is the change from baseline in quality-of-life and cost-effectiveness? (Original RCT)^b^
**Domain 4: The adopter system**		
	4A. What changes in staff roles, practices, and identities are implied?	No changes
	4B. What is expected of the patient (and/or immediate caregiver)—is this achievable by, and acceptable to, them?	No changes
	4C. What is assumed about the extended network of lay caregivers?	Not applicable
**Domain 5: The organization**		
	5A. What is the organization’s capacity to innovate?	No changes
	5B. How ready is the organization for this technology-supported change?	No changes
	5C. How easy will the adoption and funding decision be?	No changes
	5D. What changes will be needed in team interactions and routines?	No changes
	5E. What work is involved in implementation and who will do it?	No changes
**Domain 6: The wider context**		
	6A. What is the political, economic, regulatory, professional (eg, medicolegal), and sociocultural context for program rollout?	No changes
**Domain 7: The time dimension**		
	7A. How much scope is there for adapting and coevolving the technology and the service over time?	No changes
	7B. How resilient is the organization to handling critical events and adapting to unforeseen eventualities?	No changes

^a^RCT: randomized controlled trial.

^b^We already published the results of this measure in the original RCT [5].

## Results

A summary of the challenges of the IBD-live telemonitoring strategy per domain is given in Table 3.

In the following sections, we give a detailed overview of the considerations we have made during the rating process.

### Domain 1: The Illness

Patients were followed for 52 weeks. During this observation period, 448 Flarometer scores were generated, of which 176 (39.3%) were in the green stratum, 181 (40.4%) were in the orange stratum, and 91 (20.3%) were in the red stratum. Overall, 30 of 176 green *all-is-well* results were generated during a close-out visit and were excluded from analysis. The median change in fecal calprotectin concentration in the follow-up stool of the 146 remaining cases was 0 (interquartile range, IQR 0-70) ug/g (Multimedia Appendix 1). A total of 11 of 91 red *out-of-range* alerts were generated during a close-out visit and were excluded from analysis for not leading to a response from patient or health provider anymore. Among the remaining 80 red alerts were the patients who were most likely to benefit from an encounter with their specialist because of an imminent flare. In 39 of 80 cases, the local IBD team followed the automated advice to intensify treatment. The median change in fecal calprotectin concentration in the follow-up stool in this group was −255 (IQR −975 to 20) ug/g. In 21 of 80 cases, there was a well-founded reason to deviate from the treatment advice and follow a watchful waiting strategy. These reasons included the presence of colon pathogens and high symptom scores caused by disorders that were not related to IBD such as irritable bowel syndrome. In these cases, the median change in fecal calprotectin concentration in the follow-up stool was −216 (IQR −1965 to 0) ug/g. In 20 of 80 red alerts, there was no contact between patient and health provider, despite 3 email notifications and an attempt to contact the patient by telephone after 2 weeks of passivity. This strategy violation led to a median change in fecal calprotectin concentration of 167 (IQR 0-1047) ug/g. Multimedia Appendix 1 shows that the direction of change in calprotectin concentration in the latter group was different from the others and toward further deterioration.

The foregoing made us decide that ad hoc face-to-face follow-up consultations were fairly predictable and consistent in the group with out-of-range results. We had few unpredictable eventualities in the all-is-well results. Both disease relapses and sustained remissions were predictable, and the treatment advice generated by the Flarometer was reliable. This measure was therefore classified as *simple*.

### Domain 2: The Technology

The telemonitoring strategy relied on calprotectin measurements in stool samples that were sent to the hospital laboratory by ordinary mail. Immediately after arrival, the stool sample was analyzed with a point-of-care test that offers quantitative results within minutes. This rapid test has good agreement with the established enzyme-linked immunosorbent assay (ELISA) and can therefore serve as a reliable alternative to the time-consuming ELISA in the follow-up of patients with IBD [10]. We plotted the calprotectin results per patient over time to give both patient and doctor an overview of the inflammatory state of the gut during the experiment. Patients found the longitudinal tracking of calprotectin results helpful and trusted the results. However, doctors were equivocal about whether a rise in calprotectin above 250 ug/g was sufficient to justify treatment intensification. Some wished to perform additional diagnostic tests to rule out gastrointestinal infections before progressing to a treatment decision. Multimedia Appendix 2 shows that concurrent gastrointestinal infections were found in 14% of stools with out-of-range results, and this proportion was not statistically different from stools with calprotectin values in the target range (below 250 ug/g).

Use of the telemonitoring strategy required no previous knowledge from the patients except for recognition of what conditions count as urgent, such as a drug reaction. All measures within this domain were classified as *simple*, with the exception of 2D: the sequence of events from sending an automated reminder by email to the formulation of a treatment advice (Figure 1), relying on a bespoke solution from a small-sized enterprise, which carries a risk of supplier withdrawal. However, its relatively substitutability is relatively straightforward, and the complexity of this measure was therefore classified as *complicated*.

### Domain 3: The Value Proposition

Our telemonitoring strategy depended on a predictive algorithm on the basis of calprotectin changes over time to prevent an anticipated disease flare and avert unplanned outpatient visits and day care procedures (including colonoscopies). Although the technology created new possibilities for *personalized* medicine, such as selecting and targeting the patients who are most likely to benefit from a face-to-face encounter with their specialist, we did not observe a reduction in workload. Patients assigned to the telemonitoring group had fewer face-to-face encounters with the IBD team compared with those in conventional follow-up, but the number of telecontacts (emails and telephone calls) increased by 40%. The telemonitoring strategy was cost effective, as it demonstrated comparable effectiveness with conventional follow-up at lower costs. In the intention-to-treat analysis, telemonitoring led to a mean annual cost saving of €89 per patient, and this amount increased to €360 in those who were adherent to the technology. The time-saving aspect of the technology and the better sense of disease control were highly valued by patients and their caretakers. Not all patients were suitable to be followed in a telemonitoring program. It is important to realize that differences exist in people’s desire for information. Some patients seek as much information as possible about the threat of a disease flare (*monitors*), whereas others try to avoid potentially threatening information (*blunters*) [11]. The Flarometer technology will give the former category of patients and parents a greater sense of control, as opposed to the latter category who may feel more vulnerable because of the constant confrontation with their chronic disease. This aspect may partially explain attrition in the telemonitoring arm.

We are not sure that use of the telemonitoring strategy reduces the demand on health services, but it certainly allows selecting and targeting the patients who are most likely to benefit from a face-to-face encounter with their specialist. Furthermore, the business case is still underdeveloped. We therefore classified measure 3A as *complicated*. However, the technology itself is desirable for patients, and it is safe and cost effective, particularly in those who are adherent to the telemonitoring strategy, and measure 3B was therefore classified as *simple*.

**Table 3:** Summary of the challenges of the *IBD-live* telemonitoring strategy based on the nonadoption, abandonment, scale-up, spread, and sustainability framework.

Domain and question		Rating^a^	Explanation
**Domain 1: The illness**			
	1A. How well does the Flarometer strategy predict out-of-range or all-is-well events?	+++	Ad hoc face-to-face follow-up consultations were fairly predictable and consistent in the group with out-of-range results; few unpredictable eventualities in the all-is-well results.
	1B. What are the sociocultural factors associated with good adherence to the technology?	+++	Majority of teenage patients were considered *suitable* for the technology, except for those with concurrent diseases such as arthritis and sclerosing cholangitis; Patients with delayed emotional maturity, prominent functional gastrointestinal disease, or with little knowledge of the Dutch language were considered *less suitable* for the technology.
**Domain 2: The technology**			
	2A. Perceived usability	+++	Access to Web-based portal was easy via a link in the email notification. The tunnel design (Figure 1) guided the patients in a few steps in sequential order through the Flarometer. Successful stool-based monitoring system for teenagers heavily relies on active and coordinating role of the parent.
	2B. Appropriateness of the automated treatment advice	+++	Treatment advice was accepted and trusted by patients. Concurrent gastrointestinal infection was found to be the cause of the out-of-range result in a minority of cases.
	2C. Knowledge and/or support required to use the technology	+++	Use of the technology requires no previous knowledge from the patient, except for recognizing what conditions count as urgent.
	2D. Technology supply model	++	Technology relies on bespoke solution from a small-sized enterprise with risk of supplier withdrawal.
**Domain 3: The value proposition**			
	3A. Developer’s business case for the technology (supply-side value)	++	Not sure that use of the technology reduces the demand on health services, but it certainly allows selecting and targeting the patients who are most likely to benefit from a face-to-face encounter with their specialist.
	3B. Efficacy, safety, and cost-effectiveness of the technology (demand-side value)	+++	Technology is desirable for patients, it is safe and cost effective, particularly in those who are adherent to the telemonitoring strategy.
**Domain 4: The adopter system**			
	4A. Implications for staff roles, practices, and identities in case of adoption	+++	Difference between innovators and early adopters (who embraced the technology) on 1 side and a minority of other health providers (who were reluctant to adopt the technology). Technology was not seen as a threat to job security.
	4B. What is expected of the patient (and/or immediate caregiver)—and is this achievable by, and acceptable to, them?	+++	Patients were already familiar with the stool collection procedure. Logging on to the system was easy. The study presupposed that parents or carers were actively supporting their child during the study observation period.
**Domain 5: The organization**			
	5A. Organization’s capacity to innovate	+	Technology follows the natural work flow but conflicts with established hospital electronic databases and therefore requires double data entry. This will put a strain on the already overstretched health service.
	5B. Readiness for this technology-supported change	++	No linked routine for booking face-to-face appointments in case of a red alert.
	5C. Easiness of adoption and funding	++	Anticipated reduction in costs were not realized as case management was not always successful in avoiding follow-up consultations and day care admissions. Neutral cost-benefit balance.
	5D. Changes required in team interactions and routines	++	Variation in clinician engagement was based on the vision of local teams of whether remote biomarker monitoring enhances rather than threatens the existing service. Expansion of *rapid access* possibilities is required.
	5E. Work and persons involved in implementation	+	Significant work needed to build shared vision, engage staff, enact new practices, and monitor impact.
**Domain 6: The wider context**			
	6A. Political, economic, regulatory, professional, and sociocultural context for program rollout	+++	Effect of January 2018, the Dutch Health care Authority has agreed that screen-to-screen consultations will be reimbursed at a rate equivalent to face-to-face consultations, provided that a substantive report is added to the patient’s medical record.
**Domain 7: The time dimension**			
	7A. Scope for adapting and coevolving the technology over time	++	The technology can easily be adapted over time.
	7B. Handling critical events and adaptation to unforeseen eventualities	+++	The research head quarter and the Web designer were able to detect critical events quickly and respond to these through coordinated action.

^a^Rating: Simple +++; Complicated ++; Complex +.

### Domain 4: The Adopter System

**Figure 3:**
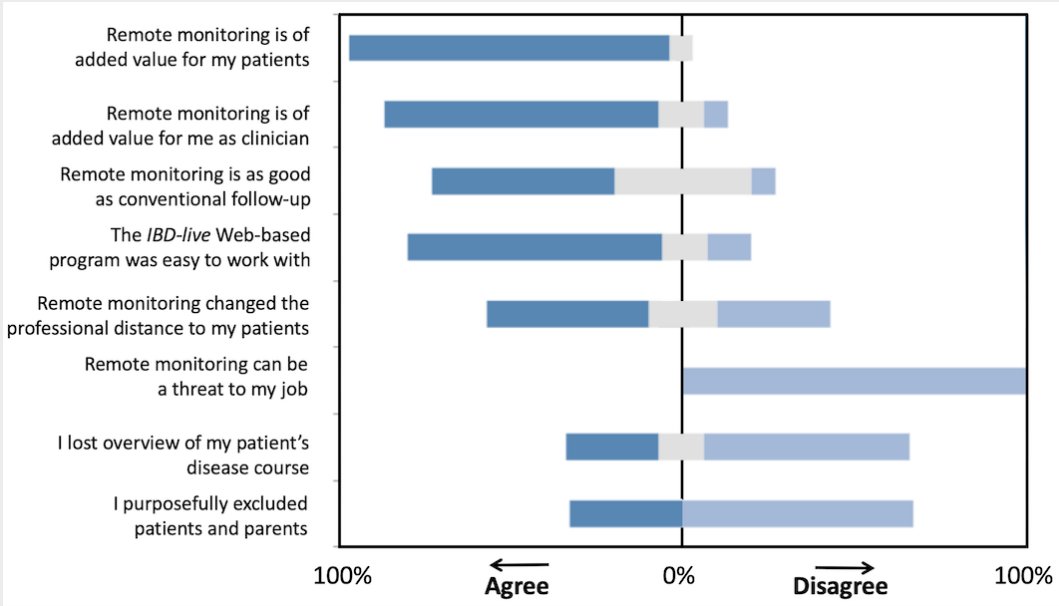
Health provider’s opinions about home telemonitoring (n=15). The proportion of respondents who agreed to the statements (left of the neutral line) versus those who disagreed (right of the neutral line). IBD: inflammatory bowel disease.

The structured questionnaire that was spread among health providers after termination of the RCT revealed the difference between innovators and early adopters (who embraced the technology) on 1 side and a minority of other health providers who were reluctant to adopt the technology on the other. The hurdle was not learning to use a new technology but rather the prospect of double data entry when running a telemonitoring service in parallel with conventional follow-up. This perception of overload will put a strain on already overstretched health services. The telemonitoring strategy was not seen as a threat to job security, although a considerable proportion of health providers felt that it changed the professional distance to their patients (Figure 3).

For patients, the easiest way to log on to our system was via a link in the email notification. Some patients forgot their username or password, and the time involved in recovering their username or resetting their account password demoralized them. The tunnel design guided the patients in a few steps in sequential order through the Flarometer, and entering data was merely clicking in a data entry form. The patients were already familiar with the stool collection process with its inherent obstacles [12].

As neither existing staff nor patients and caregivers had to learn new skills, we classified all measures within this domain as *simple*.

### Domain 5: The Organization

Decision makers may be disappointed to read that cost-savings by the use of telemonitoring were only modest, but physician-nurse task shifting, on the other hand, appeals greatly to health decision makers. As the technology allows to select and target patients who benefit from a face-to-face encounter with their physician at short notice for timely adjustment of therapy plans, nurse specialists in IBD treatment teams may dedicate their time to other problems, including repeat prescriptions, quality-of-life issues, and absenteeism from school or work, not to mention that time will be needed to motivate patients who ignored automated reminders. Successfully accomplishing a task shift probably involves the expansion of the number of rapid access clinic consultation slots. In addition, currently, there is no built-in routine for booking rapid consultations in case of out-of-range results. Although the telemonitoring strategy follows the natural work flow, it conflicts with established hospital electronic databases and therefore requires double data entry. This will put a strain on already overstretched health services. To reduce double documentation, clinicians may want to import data from electronic health records into the cloud-based telemonitoring system or vice versa. This is currently not possible. The organization was perceived as the most problematic of all domains. We classified measures 5A and 5E as *complex*. The last question in domain 5 concerns the work involved in implementation. We regret to say that such a study is extensive and often hidden, and we typically underestimated it at the planning stage. Measures 5B to 5D were classified as *complicated*.

### Domain 6: The Wider Context

The most significant system-level challenge at the time that we performed our study was the absence of an established national tariff for reimbursing electronic health consultations. In the meantime, the Dutch Health care Authority agreed that with effect from January 2018, screen-to-screen consultations are reimbursed at a rate equivalent to face-to-face consultations, provided a substantive report is added to the patient’s medical record [13]. Reimbursement is only possible when the screen-to-screen consultation is really comparable in both content and duration with a regular face-to-face consultation at the clinic. Second, the screen-to-screen contact should be booked in advance. As financial and regulatory requirements are now in place nationally, we classified this measure as *simple*.

### Domain 7: The Time Dimension

We used email notifications to communicate with the target population, but teenagers hardly use this *ancient form* of digital communication anymore. Despite being an over 40-year-old technology, email has many advantages to newer ways of communication such as WhatsApp, and it is still considered a more trustful way of communication. Email is persistent, easily searchable, one is able to send it from virtually every internet-able device in the world, it uses small data volume, one can attach whatever is wanted, and it is expandable (adding encryption or signing for instance). Having said all this, better, safer, and more appealing tools for communication in health care are a good thing. It will be interesting to see what swoops in to fill the gaps for patient-related data exchange in health care.

As the potential for adapting and coevolving the technology and service over time is uncertain, we classified this measure as *complicated*. With the technology that we used during the RCT, the research head quarter and the Web designer were able to detect critical events quickly and responded to these through coordinated action, therefore question 7B was classified as *simple*.

## Discussion

### Principal Findings

We recently reported on a telemonitoring strategy for teenagers with IBD (*IBD-live*) that was designed for early detection of relapse and follow-up of appropriateness of treatment. We concluded that the technology was a safe and cost-saving alternative to conventional follow-up. For patients and parents in the intervention arm, the time-saving aspect and the better sense of disease control were highly valued. In this empirical case study, we used the newly developed NASSS framework to explore the challenges to implementing the *IBD-live* strategy and decide whether we should abandon this technology or sustain it within existing sites [9]. The framework allowed us to explore complexity in multiple interacting domains. Aspects of the different domains were classified as simple (+++: straightforward, predictable, and with few components—as in making a sandwich), complicated (++: predictable, but with multiple interacting components or issues—as in building a rocket), or complex (+: dynamic, unpredictable, and not easily disaggregated into constituent components—as in raising a child). Our technology-supported telemonitoring strategy was designed to recognize and manage disease flares at a distance, and following the treatment advice of the Flarometer led to similar patient outcomes as the doctor’s advice. This observation led to the conclusion that the condition that we aim to monitor (domain 1) is well characterized, well understood, and predictable (in other words, simple). The technology that we used (domain 2) was user-friendly for both patients and doctors, but a minority of health providers did not trust the treatment advice generated by the Flarometer. As colon pathogens were only occasionally detected in the stool samples with out-of-range results, we feel that their apprehension may not be well founded. The Flarometer algorithm relied on a bespoke solution from a small-sized enterprise, which always carries a risk of supplier withdrawal. However, its substitutability is relatively straightforward, and this aspect of the technology was therefore classified as *complicated*. For patients, parents, and health providers, the value of telemonitoring (domain 3) is in the possibility for *personalized* medicine, such as selecting and targeting the patients who are most likely to benefit from a face-to-face encounter with their specialist. Access to care was improved in those patients who completed the Flarometer from their home with a result in the low-risk stratum and were reassured without missing school or work, whereas those with an out-of-range result were triaged by an IBD nurse for a physical office visit. However, in a busy, appointment-based practice, scheduling an urgent patient can be a challenge. Organizations that wish to use the telemonitoring strategy to the fullest may want to think of a flexible system to cater for urgent patients, for example, by expanding the number of rapid access clinic consultation slots. The supply-side value was therefore not merely classified as *simple* but rather as *complicated*.

The greatest challenge to scale-up, spread, and sustain our telemonitoring strategy is, without a doubt, the organization’s capacity (domain 5) to build the new technology into this workflow, for example, by automating the steps for booking office visits in case of suspected disease flares.

### Particular Strengths and Limitations of the Project

The main strength of our telemonitoring project was its randomized and pragmatic design. A heterogeneous group of patients, parents, health providers, and clinical practices were included to maximize the applicability of the results to everyday practice. IBD-live was developed through close collaboration among developers, clinicians, and representatives of the Dutch Crohn’s and Colitis patient organization (CCUVN). The director of the CCUVN was consulted at the inception stage of the project and was also on the committee of reviewers who decided on the ZonMw funding.

However, this empirical case study shows that a sturdy evidence base for the efficacy of the intervention does not guarantee a spontaneous movement of the intervention into routine clinical usage. Historically, this research-to-practice gap has not been the concern of academic clinical researchers [14]. We used the NASSS framework retrospectively and were only able to explain why we failed to implement telemonitoring in our daily clinic till now. If we would have used the framework at an early stage of our telemonitoring program development, it would have saved us precious time. In the future, we will definitely use this framework prospectively and in real time to support new programs, and we also encourage others to do so. This case study hinges on the rating of challenges summarized in Table 3. Although 2 assessors (AD and PFvR) independently used a rating matrix, the generalizability of our findings is problematic. There is a great deal of subjectivity in how the challenges were rated, which may have affected the conclusions of this paper.

### Comparison With Other Studies

In contrast with our telemonitoring strategy that is not yet mainstreamed, we know of 1 Dutch IBD telemonitoring initiative that has been successfully implemented—myIBDcoach [15,16]. This telemedicine system monitors and registers disease activity in patients with all subtypes of IBD as well as patient-reported outcome measures and quality metrics. We have tried to comprehend why this initiative, which focuses on adult patients with IBD, has a broad support and is expanding to both academic and nonacademic IBD centers. The most important difference with our own project relates to the institution of a foundation to facilitate implementation and to improve cooperation among the various stakeholders. The director of the Dutch IBD patients’ organization, 2 gastroenterologists, an accountant, and an assistant professor of health analytics systems constitute the board. Representatives of the IBD section of the Dutch Association for Gastroenterology, the Dutch Association for Gastroenterologists, and the Association of IBD Nurse Specialists form a separate committee which decides on the design and content of myIBDcoach.

Several other telemonitoring initiatives in IBD care, with direct connection to the IBD specialist team, have been developed in Denmark, United States, and the United Kingdom [17-21]. However, in spite of the promising results in the field of responsive and cost-effective care, these initiatives are all in the stage of sustained adoption and none are ready for distant spread.

### Interpretation

This case study illustrates that our system with remote biomarker monitoring enhances rather than threatens the existing hospital service with periodic office visits. Home telemonitoring is attractive for teenagers and their families, and health professionals may be interested in using it to keep teenagers who are well out of hospital and ease the pressure on overstretched outpatient services. The telemonitoring system is ideally run with IBD nurses to provide full coverage during working hours. Patients with disease in remission may have up to 4 Flarometer measures in 1 year and 1 routine physical office visit. Patients with an anticipated disease flare will be booked in between for a rapid access clinic consultation. Patients who require periodic day care infusions with antitumor necrosis factor (TNF) monoclonal antibodies may not profit from the telemonitoring service as they will already have unavoidable frequent contact with health care providers. However, those on injectable anti-TNFs, may also highly value our telemonitoring strategy. Home telemonitoring can move IBD care into a new era in which teenagers take ownership of their chronic disease and participate in the therapeutic decision-making process on the basis of longitudinal tracking of Flarometer results. It is important to realize that a wide range of services should be available, as patients and their families have different needs related to both disease activity and personal circumstances. Individual needs also change over time, which require a flexible contact to the hospital.

### Abandon or Ready for Sustained Adoption?

Application of the NASSS framework has shown what issues will be mission-critical to move our telemonitoring strategy from a demonstration project to one that is fully mainstreamed and part of business as usual locally. The challenges to be addressed are merely *complicated* (++, ie, predictable and controllable) rather than *complex* (+), which means that our program may be difficult but not impossible to sustain within existing sites.

On the basis of the NASSS framework, we conclude the following:

- The technology and linked program were designed around a well predictable condition (disease flare) in patients with IBD.

- The prototype of the technology was sufficiently tested in an RCT.

- The value proposition of the technology was clear, with a distinct benefit for patients and an affordable service model.

- Health providers have plausible personal reasons to resist (double data entry).

- The organization may not be ready for the innovation, as it requires a shift to new ways of working.
- The concerns about reimbursement are not valid reasons to stall the mainstreaming of the technology.

- The technology is able to adapt and evolve over time in a way that continues to meet the needs of its users.

After integrating the technology and its use with local workflows, we believe that our telemonitoring strategy will be ready for distant spread and long-term sustainability.

## Appendix

Multimedia Appendix 1 Median (inter quartile range, IQR) change in fecal calprotectin. The time interval after a green all-is-well result and a red out-of-range alert was 4 and 2 months respectively. A positive value represents an increase in fecal calprotectin.

Multimedia Appendix 2 Presence of colon pathogens in stool samples as per flarometer color code.

## Abbreviations

CCUVN: Dutch Crohn’s and Colitis patient organization
ELISA: enzyme-linked immunosorbent assay
IBD: inflammatory bowel disease
IQR: interquartile range
NASSS: nonadoption, abandonment, scale-up, spread, and sustainability
RCT: randomized controlled trial
TNF: tumor necrosis factor
