# Supplementary figures and images for: Exploring the Challenges of Implementing a Web-Based Telemonitoring Strategy for Teenagers With Inflammatory Bowel Disease: Empirical Case Study
Source: J Med Internet Res. 2019 Mar 29;21(3):e11761. doi: 10.2196/11761 (PMC6460310; doi:10.2196/11761)

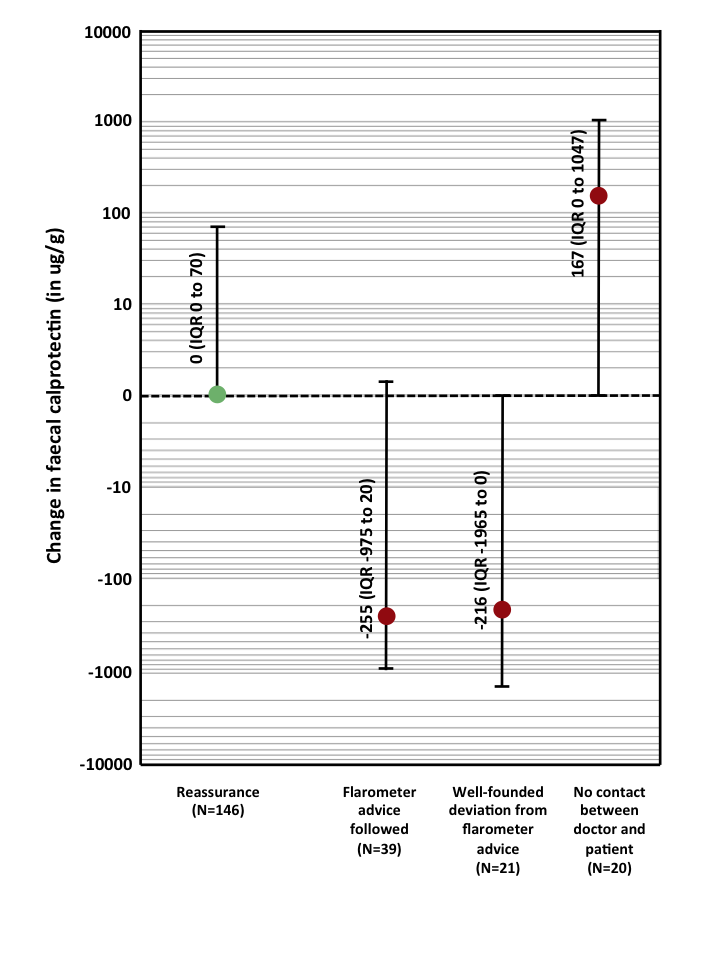

Supplement: Multimedia Appendix 1 [file jmir_v21i3e11761_app1.png]

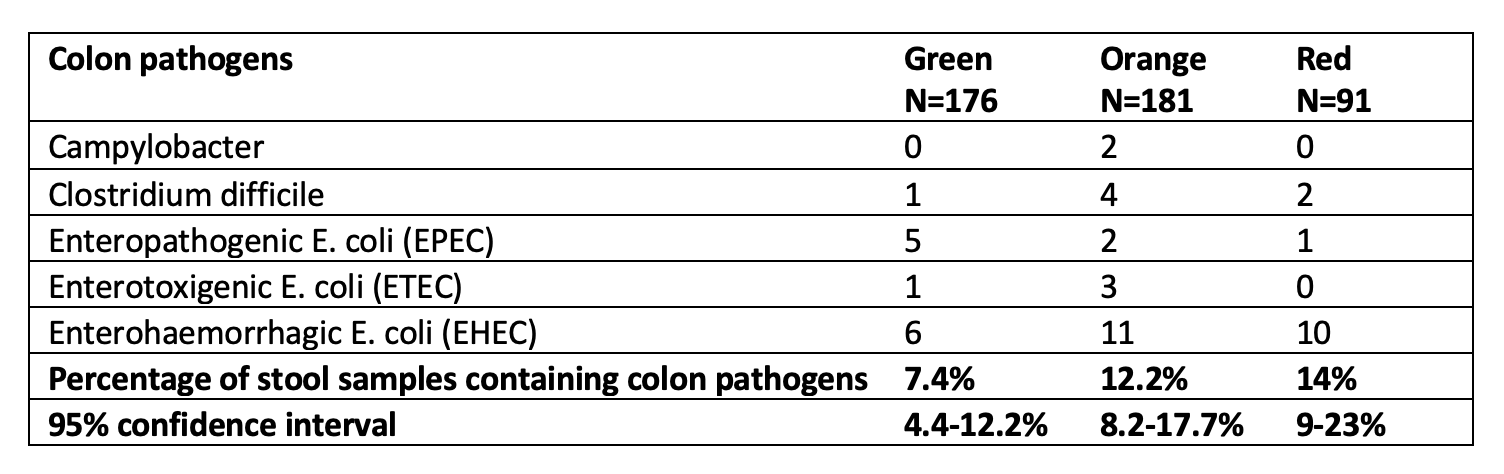

Supplement: Multimedia Appendix 2 [file jmir_v21i3e11761_app2.png]
